# Gene and protein expression of *O*-GlcNAc-cycling enzymes in human laryngeal cancer

**DOI:** 10.1007/s10238-014-0318-1

**Published:** 2014-10-15

**Authors:** Katarzyna Starska, Ewa Forma, Ewa Brzezińska-Błaszczyk, Iwona Lewy-Trenda, Magdalena Bryś, Paweł Jóźwiak, Anna Krześlak

**Affiliations:** 1 I Department of Otolaryngology and Laryngological Oncology, Medical University of Łódź, Kopcinskiego 22, 90-153 Lodz, Poland; 2 Department of Cytobiochemistry, University of Łódź, Pomorska 142/143, 90-236 Lodz, Poland; 3 Department of Experimental Immunology, Medical University of Łódź, Pomorska 251, 92-213 Lodz, Poland; 4 Department of Pathology, Medical University of Łódź, Pomorska 251, 92-213 Lodz, Poland

**Keywords:** *O*-GlcNAc, OGT, OGA (*MGEA5*), Tumor front grading (TFG), Laryngeal cancer

## Abstract

Aberrant protein *O*-GlcNAcylation may contribute to the development and malignant behavior of many cancers. This modification is controlled by *O*-linked β-*N*-acetylglucosamine transferase (OGT) and *O*-GlcNAcase (OGA). The aim of this study was to determine the expression of *O*-GlcNAc cycling enzymes mRNA/protein and to investigate their relationship with clinicopathological parameters in laryngeal cancer. The mRNA levels of *OGT* and *MGEA5* genes were determined in 106 squamous cell laryngeal cancer (SCLC) cases and 73 non-cancerous adjacent laryngeal mucosa (NCLM) controls using quantitative real-time PCR. The level of OGT and OGA proteins was analyzed by Western blot. A positive expression of *OGT* and *MGEA5* transcripts and OGT and OGA proteins was confirmed in 75.5 and 68.9 % and in 43.7 and 59.4 % samples of SCLC, respectively. Higher levels of mRNA/protein for both OGT and OGA as well as significant increases of 60 % in total protein *O*-GlcNAcylation levels were noted in SCLC compared with NCLM (*p* < 0.05). As a result, an increased level of *OGT* and *MGEA5* mRNA was related to larger tumor size, nodal metastases, higher grade and tumor behavior according to TFG scale, as well as incidence of disease recurrence (*p* < 0.05). An inverse association between *OGT* and *MGEA5* transcripts was determined with regard to prognosis (*p* < 0.05). In addition, the highest OGT and OGA protein levels were observed in poorly differentiated tumors (*p* < 0.05). No correlations with other parameters were noted, but the results showed a trend of more advanced tumors to be more frequently OGT and OGA positive. The results suggest that increased *O*-GlcNAcylation may have an effect on tumor aggressiveness and prognosis in laryngeal cancer.

## Introduction

*O*-GlcNAcylation is a reversible posttranslational modification (PTM) that regulates the activity of nuclear, cytoplasmic and mitochondrial proteins and modifies a wide range of fundamental cellular processes and functions. This *O*-linked β-d-*N*-acetylglucosamine (*O*-GlcNAc) modification of the hydroxyl group of serine and/or threonine residues in proteins is emerging as a crucial regulator of such biological mechanisms as transcription, translation, protein stability, proteasomal degradation, apoptosis and signal transduction in response to cellular and environmental cues, such as nutrient fluxes, growth factors, signaling molecules, hormones and stress [1–4]. The inducible cycling of this modification in highly proliferating cells is dynamically managed by two enzymes, *O*-GlcNAc transferase (OGT) and *O*-GlcNAcase (OGA, encoded by *MGEA5* gene), which, respectively, catalyze the addition and removal of a single *N*-acetylglucosamine (GlcNAc) molecule in response to UDP-GlcNAc levels in the hexosamine biosynthetic pathway (HBP), a minor branch of glycolysis [1–4].

A literature study highlights the contribution of aberrant *O*-GlcNAcylation of proteins to the pathological progression of metabolic diseases such as diabetes, heart diseases, neurodegenerative disorders and cancer [1, 5–9]. An increasing body of evidence indicates that *O*-linked β-d-*N*-acetylglucosamine modification may play an important role in tumorigenesis and redirection of cancer metabolism to the less efficient glycolytic pathway, known as the Warburg effect, but direct functional connections have until now remained poorly defined [6–9].

In the last half decade, research has been focused on the identification of mechanisms through which *O*-GlcNAcylation might induce a malignant cell phenotype and determine cell proliferation, growth, tumor invasion and survival when acting at specific sites on such proteins as key transcription factors, metabolic enzymes and oncogenic signaling pathway molecules, altering their cellular functions [6–9]. *O*-GlcNAcylation has been shown to play a critical role in carcinogenesis and cancer development through different mechanisms, especially by modulating the expression and/or activity of major oncogenic factors (MYC, p53, NF-κB, FOXM1), impacting tumor cell adhesion and migration by the regulation of the E-cadherin/β-catenin system, and by promoting the expression of metalloproteins [3, 6–10]. For example, experimental evidence has confirmed that the proto-oncogene MYC can be *O*-GlcNAcylated on Thr58, the site normally phosphorylated by GSK3β in response to MYC phosphorylation on Ser62 resulting in MYC degradation. Hyper-*O*-GlcNAcylation is able to stabilize MYC and promote its higher activity [11]. In a similar way, *O*-GlcNAcylation at Ser149 of a wild-type p53 prevents the phosphorylation at Thr155 by the COP-9 signalosome and inhibits p53 degradation. This modification favors proto-oncogenic p53 mutant stability and its tumor suppressor activity [12, 13]. *O*-GlcNAc modification is also known to regulate NF-κB transcription factor signaling. These hypothetical mechanisms involve both *O*-GlcNAcylation of the IKKβ kinase on Ser733, which prevents IKKβ inhibition and results in sustained activity responsible for the pro-oncogenic NF-κB status and *O*-GlcNAcylation at the Thr322 site, and Thr352, the central residue for transcriptional activation of NF-κB in presence of high glucose concentration [14, 15]. Numerous studies in vivo and in vitro suggest that *O*-GlcNAc promotes the epithelial-mesenchymal transition (EMT), cell migration, invasion and metastasis [16–18]. More recently, decreased expression of matrix metalloproteinases (MMPs) and VEGF level upon OGT inhibition has been demonstrated [19, 20].

The clinical importance of alternations in *O*-GlcNAc signaling and aberrant expressions of OGT and OGA in cancer is, unfortunately, largely unknown. Studies on different types of tumors generally indicate higher level of *O*-linked β-d-*N*-acetylglucosamine modification and changes in OGT and/or OGA levels, but divergent results concerning *O*-GlcNAc cycling enzymes can be found [11, 14, 20–28]. A literature review reveals that cancer cells and tissues appear to display overexpression of both, OGT and *O*-GlcNAc modification in most cases. The data indicate that higher OGT and *O*-GlcNAcylation levels are related to enhanced grade of tumor aggressiveness, higher incidences of metastasis as well as poor prognosis in prostate, breast, endometrial, colorectal, pancreatic, ovarian, lung, bladder cancers and chronic lymphocytic leukemia cells [11, 14, 20–26]. However, these findings have not been confirmed, and other studies indicate that variable and diverse aberrations of *O*-GlcNAc cycling enzyme activity exist in primary tumors and matched adjacent tissues [17, 27, 28]. Despite conflicting data, research reveals that identification of changes in both OGT and OGA expressions, as parameters of cancer metabolism reprogramming, and oncogenic mechanisms leading to increased *O*-GlcNAc levels may be considered as novel indicators of tumor invasiveness and prognostic biomarkers of various types of cancer. They may also represent potential new therapeutic targets for treatment strategies, also in synergy with conventional treatments, to alter and/or inhibit cancer development and progression. Unfortunately, a similar literature review reveals no publication describing the activity of *O*-GlcNAc transferase and *O*-GlcNAcase and their relationship with tumor aggressiveness and clinical behavior in head and neck carcinomas. Therefore, to identify new biomarkers of an invasive tumor phenotype, which may also constitute a potential new approach for laryngeal cancer treatment, it is necessary to assess the level of *O*-GlcNAc cycling enzymes in the most frequent cancers of head and neck origin, also with respect to indication of OGT and OGA.

The aim of this study was to determine the mRNA expression of genes coding *O*-GlcNAc transferase (*OGT*) and *O*-GlcNAcase (*MGEA5*) and their corresponding proteins, as well as to analyze total protein *O*-GlcNAcylation levels in cancerous tissue compared with matched adjacent normal tissue. In addition, the study also investigates the effect of changes in *O*-GlcNAc-cycling enzyme expression on tumor invasiveness and patient prognosis in laryngeal cancer.

## Materials and methods

### Patients and samples

In this study, 106 fresh biopsy tissue samples obtained from squamous cell laryngeal carcinoma (SCLC) cases were investigated. The patients (100 male and 6 female, mean age 62.4 ± 9.1 years) were recruited between January 2003 and December 2011 and were under treatment at the Department of Otolaryngology and Laryngological Oncology, Medical University of Łódź, Poland. All individuals had a confirmed diagnosis of SCLC based on histopathological evaluation and had undergone partial or total laryngectomy, depending on the extent of neoplastic lesions. The control group constituted 73 tissue samples from a morphologically estimated non-cancerous laryngeal mucosa (NCLM) from individuals who were qualified for total laryngectomy. According to ethical rules, the adjacent normal epithelium of the larynx in patients qualified for partial laryngectomy was not sampled. Normal laryngeal tissues were collected from the sites as far as possible from the margins of the tumor by individually harvesting samples from presumptive non-cancerous regions. All fresh samples were stored at −80 °C before analysis. The tissue specimens collected in the operation room were prepared and evaluated by an experienced pathologist. H&E-stained sections provided histological confirmation of non-cancerous and cancer tissues. The criteria for patient participation in this study were as follows: (1) A pathologically confirmed diagnosis of *squamous cell planoepitheliale carcinoma*, (2) primary surgical resection without receiving prior immuno-, radio- or chemotherapy, (3) absence of distant metastases and second primary neoplasms, (4) a negative history of previously diagnosed with other types of primary cancers, and (5) a negative history of recurrences of laryngeal cancer. Informed consent was obtained from each subject. The investigations were performed with the approval of the Bioethical Commission of the Medical University of Łódź and the National Science Council, Poland (approval No RNN/60/13/KE). In all cases, surveys were performed to complete the cancer registry database. The database catalog was queried every 2 months and identified all histopathologically confirmed incident primary squamous cell laryngeal cancer cases reported within 4 months of diagnosis preceding the recruitment. The socio-demographic features of the study subjects are presented in Table 1.

**Table 1:** Socio-demographic characteristics of study subjects

Variable	Cases *n* (%)
Sample size	*n* = 106
Age, years	
<50 years	33 (31.1)
≥50 years	73 (68.9)
Sex	
Male	100 (94.3)
Female	6 (5.7)
Smoking status^a^	
Current	78 (73.6)
Former	23 (21.7)
Never	5 (4.7)
Alcohol intake^b^	
Never/rare	3 (2.8)
Light	16 (15.1)
Moderate	55 (51.9)
Heavy	27 (25.5)
Ex-drinker	5 (4.7)
Surgical treatment	
Total laryngectomy	73 (68.9)
Partial laryngectomy	33 (31.1)
Neck dissection (–)	60 (56.6)
Neck dissection (+)	46 (43.4)
Selective neck dissection	42 (39.6)
Radical neck dissection	4 (13.8)
Survival	
≥5 years	69 (86.8)
<5 years	37 (13.2)
Local recurrences	
Negative	93 (87.7)
Positive	13 (12.3)
Nodal recurrences	
Negative	94 (88.7)
Positive	12 (11.3)

^a^ Smoking was grouped into “current,” “former” and “never” based on self-reported usage. Participants who reported smoking at least 100 cigarettes in their lifetime and who, at the time of survey, smoked either everyday or some days were defined as current smokers. Participants who reported smoking at least 100 cigarettes in their lifetime and who had not been smoking for at least 3 months were defined as former smokers. Participants who reported never having smoked 100 cigarettes were defined as never smokers

^b^ Never/rare, <1 unit/week; light, 1–8.9 units/week; moderate, 9–17.9 units/week; heavy, ≥18 units/week; where 1 unit = 22 g ethanol

### Histological classification and morphological features

Archival formalin-fixed paraffin-embedded tissue samples were used for the histological classification of tumors. All specimens were assessed according to the criteria conducted in accordance with the AJCC TNM classification of 2010 for laryngeal cancers [29]. Morphological estimation was performed on H&E-stained sections in the most invasive, peripheral zones of the tumor, according to tumor front grading, TFG, which is one of the most reliable pathological methods for the analysis of neoplastic progress and determination of the dynamics of tumor growth, as well as a reasonably precise prognostic factor in laryngeal carcinoma [30]. The histological evaluation considered the mode and depth of invasion as well as total TFG score. The factors were assessed in at least five different regions of the peripheral part of the tumor (magn. 200×, number of mitoses magn. 400×). Each factor was graded according to a scale ranging from 1 to 4. The numeric morphological TFG score was computed as the sum of six tumor-related features (cytoplasmic differentiation, nuclear polymorphism, number of mitoses) and adjacent stroma-related characteristics of the peripheral edge of tumor infiltration (mode of invasion, depth of invasion and plasmalymphocytic infiltration), with a maximum score of 24 points. According to the TFG total score, tumors were divided into 4 groups: 6–9, 10–13, 14–17, 18–21 and >22 TFG points. The histological grade of differentiation G was measured according to the generally accepted three-grade morphological system: G1 (well-differentiated tumor), G2 (moderately differentiated tumor) and G3 (poorly differentiated tumor). The clinicopathological characteristics of the laryngeal cancers are shown in Table 2.

**Table 2:** Clinicopathological characteristics of laryngeal cancers

Variable	Cases *n* (%)
Tumor size (pT)	
pT1	10 (9.4)
pT2	29 (27.4)
pT3	33 (31.1)
pT4	34 (32.1)
Lymph node metastases (pN)	
pN0	84 (79.2)
pN1–3	22 (20.8)
Degree of differentiation (Grade)	
G1	14 (13.2)
G2	82 (77.4)
G3	10 (9.4)
TFG total score^a^	
6–9 points	5 (4.7)
10–13 points	39 (36.8)
14–17 points	39 (36.8)
18–21 points	23 (21.7)
>22 points	0
Mode of invasion	
Well-defined borderline (1 point)	14 (13.2)
Less marked borderline (2 points)	34 (32.1)
No distinct borderline (3 points)	37 (34.9)
Diffuse growth (4 points)	21 (19.8)
Depth of invasion	
Carcinoma in situ (1 point)	5 (4.7)
Microinvasion (2 points)	35 (33.0)
Nodular into submucosa (3 points)	27 (25.5)
Deep invasion (cartilage) (4 points)	39 (36.8)

^a^ The numeric morphological TFG score was computed as the sum of six tumor-related features (cytoplasmic differentiation, nuclear polymorphism, number of mitoses) and adjacent stroma-related characteristics of the peripheral edge of tumor infiltration (mode of invasion, depth of invasion and plasmalymphocytic infiltration). The factors of TFG were assessed in at least five different regions of the peripheral part of the tumor (magn. 200×, number of mitoses magn. 400×). Each factor was graded according to a scale ranging from 1 to 4

### Total RNA extraction and cDNA synthesis

Total RNA was extracted using TRI Reagent (Sigma-Aldrich, USA) according to the manufacturer’s protocol. RNA was eluted in 20 μl RNase-free water, quantified by spectrophotometry at 260 nm, and stored at −20 °C. RNA with a 260/280 nm ratio in range 1.8–2.0 was considered as high quality. First-strand cDNAs were obtained by reverse transcription of 1 μg of total RNA using RevertAid™ First-strand cDNA synthesis kit (Fermentas International, Lithuania) following the manufacturer’s protocol.

### Real-time quantitative PCR

Real-time amplification of the cDNA was performed using TaqMan^®^ Gene Expression Assay (Applied Biosystems) according to manufacturer’s instruction. The fluorogenic, FAM labeled probes and the sequence-specific primers for *OGT, MGEA5* and internal-control *HPRT1* (hypoxanthine phosphoribosyltransferase 1) were obtained as inventoried assays (Hs00201970_m1, Hs00269228_m1 and Hs02800695_m1, respectively). Abundance of *OGT* and *MGEA5* mRNA in samples were quantified by the ΔCt method. ΔCt (Ct_gene_–Ct_*HPRT1*_) values were recalculated into relative copy number values (number of *OGT* or *MGEA5* mRNA copies per 1,000 copies of *HPRT1* mRNA).

### Western blot analysis

Tissue samples were homogenized for 3 min in Potter’s homogenizer in 10 volumes of RIPA buffer containing protease inhibitors. The homogenates were then sonicated three times for 10 s each in an ice bath, centrifuged at 3,000*g* for 10 min, and supernatant was collected. Protein content was quantified by the Lowry method. An equal amount of proteins (50 μg) of different samples was resolved by 10 % SDS-PAGE and electroblotted onto Immobilon-P transfer membranes (Millipore, Bedford, MA, USA). Within each blot were homogenate samples of normal and laryngeal cancer tissues together with a reference sample. The samples were always analyzed within one blot. The membranes were not compared with each other. The blots were incubated for 2 h with rabbit antibodies specific for OGT (Cell Signaling Technology) or OGA (Sigma-Aldrich) and mouse antibodies specific for *O*-GlcNAc (RL2, Abcam). All primary antibodies were used in 1:1,000 dilution. After being washed three times with TBS, the membranes were incubated with goat anti-rabbit conjugated with horseradish peroxidase, added at a 1:5,000 dilution. The membranes were again washed several times with TBS and proteins were visualized on X-ray film by enhanced chemiluminescence. Gel-Pro^®^ Analyzer software (Media Cybernetics Inc., USA) was used for densitometry analysis of protein bands. The integrated optical density (IOD) of the bands, in a digitized picture, was measured.

All RT-PCR reactions were repeated three times for each sample. All Western blot reactions were repeated five times for each sample.

### Statistical analysis

The statistical analyses were performed using STATISTICA version 9.0 (StatSoft, Poland). Since the levels of expression in laryngeal cancer specimens did not follow a normal distribution (Kolmogorov–Smirnov test), nonparametrical statistical tests (Mann–Whitney *U* test, Kruskal–Wallis test and the Spearman rank correlation test) were applied. The Kruskal–Wallis test with post hoc multiple comparisons was used to identify the relationship between OGT and OGA mRNA and protein expression and clinicopathological parameters. Kaplan–Meier survival analysis was performed to determine the association of *OGT* and *MGEA5* mRNA expressions with overall survival. The cutoff value was established to be the median of *OGT* and *MGEA5* mRNA levels. The survival curves were compared between two groups: high (≥median value) and low (<median value) expression using log-rank tests. Distribution of quantitative variables was described using means and standard deviations. A *p* value <0.05 was considered as statistically significant.

## Results

### *O*-GlcNAc-cycling enzymes expression and total protein *O*-GlcNAcylation levels in laryngeal cancer tissue and non-cancerous laryngeal mucosa

The mRNA expression of genes coding *O*-GlcNAc-cycling enzymes in laryngeal cancer tissue (SCLC) and non-cancerous laryngeal mucosa (NCLM) was estimated by real-time quantitative PCR analysis with the *HPRT1* gene applied as a reference. Positive expressions of *OGT* and *MGEA5* transcripts was observed in 80 of 106 (75.5 %) and 73 of 106 (68.9 %) laryngeal cancer samples, as well as in 42 of 73 (57.5 %) and 29 of 73 (39.7 %) normal laryngeal tissue samples, respectively. Thus, positive expression of *OGT* and *MGEA5* in SCLC was more frequently observed in SCLC than in NCLM. The mean mRNA expression of both genes was also higher in laryngeal cancer than in the adjacent normal laryngeal tissue. A significant difference in *OGT* mRNA levels was observed between the neoplastic samples and normal tissues (*p* = 0.026). A higher level of *MGEA5* transcripts was also found in tumor specimens compared with non-cancerous surrounding mucosa, but the result was of borderline significance (*p* = 0.054). The mean mRNA expressions of *OGT* and *MGEA5* in SCLC and NCLM, as well as the results of the statistical analysis are shown in Fig. 2a. The results of the Spearman rank correlation showed that *OGT* and *MGEA5* transcript levels in laryngeal cancer were positively correlated (*r* = 0.39, *p* = 0.001).

The expression of *O*-GlcNAc-cycling enzymes at the protein level was also examined using Western blot in homogenate samples of both laryngeal carcinoma specimens and the non-cancerous adjacent mucosa.

**Fig. 1:**
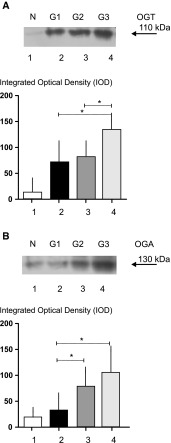
Representative immunoblot and quantitative analysis results of OGT (**a**) and OGA (**b**) protein expression in homogenate samples (50 μg protein loaded per *lane*) of normal (1) and laryngeal cancer tissues (2–4) in relation to tumor grade; lower panels show the results of quantitative densitometric analysis: 1—normal laryngeal mucosa, 2 to 4—laryngeal cancer samples classified according to the three-grade morphological system as G1 (well-differentiated tumor), G2 (moderately differentiated tumor), G3 (poorly differentiated tumor), respectively. *Graphs* represent mean ± standard deviation; *p < 0.05

A representative quantitative densitometric analysis of the intensity of *O*-GlcNAc-cycling enzyme bands from homogenates of normal and cancerous tissues is shown in Fig. 1. Of the SCLC tissue specimens, 43.8 % (14/32) and 59.4 % (19/32) showed positive OGT and OGA protein expressions, respectively. In the case of NCLM tissue, OGT and OGA protein-positive expressions were observed in 36.7 % (11/30) and in 53.3 % (16/30) samples, respectively. Mean expression of OGT and OGA proteins in cancer tissue were higher than in normal samples.

**Fig. 2:**
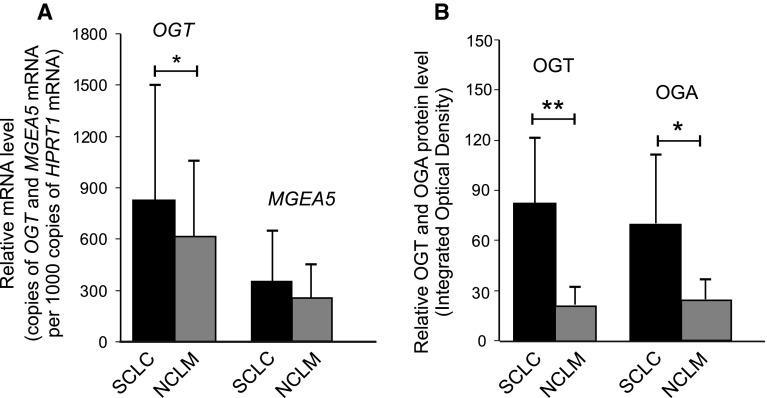
Differences between *OGT* and *MGEA5* transcript expressions (**a**) as well as between OGT and OGA protein expressions in homogenate samples (**b**) of laryngeal cancer tissue and normal laryngeal mucosa. *Graphs* represent mean ± standard deviation; *p < 0.01, **p < 0.001

A significant difference in OGT and OGA protein levels was noted in laryngeal cancer tissue compared with adjacent normal laryngeal mucosa (*p* = 0.0004 and *p* = 0.007, respectively). Mean OGT and OGA protein expressions in SCLC and NCLM as well as the results of the statistical analysis are shown in Fig. 2b. The relationship between OGT and OGA protein expressions was also analyzed, but no correlation was found (*p* > 0.05).

**Fig. 3:**
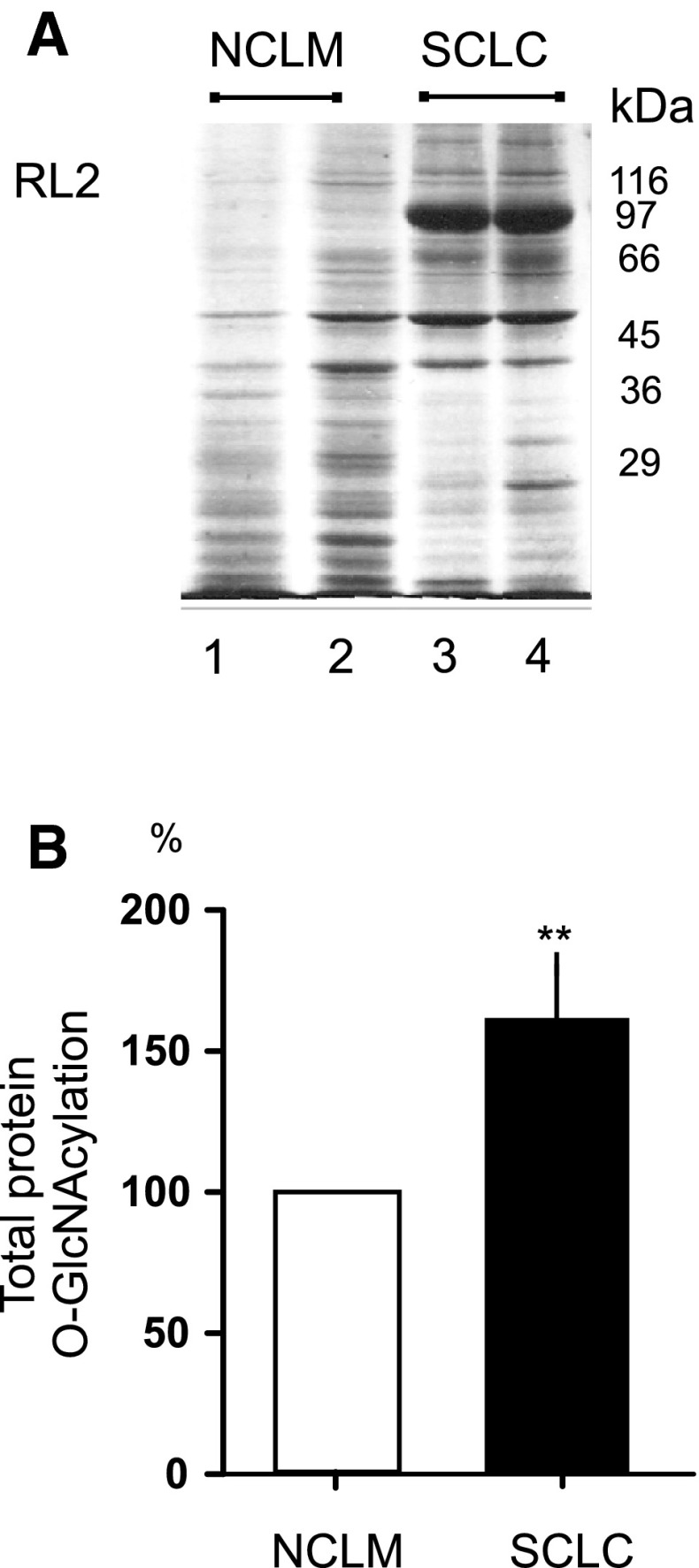
Representative image obtained from normal and laryngeal cancer sample homogenates (50 μg protein loaded per *lane*) probed with the RL2 antibody, where multiple bands were detected and used to quantify protein *O*-GlcNAcylation (**a**); plots demonstrate significant increases of ~60 % in total protein *O*-GlcNAcylation levels in laryngeal cancers tissues compared with normal tissues; **p < 0.01 (**b**)

The next stage also investigated whether the changes in OGT and OGA activities may affect total protein *O*-GlcNAcylation levels in SCLC compared to NCLM. The laryngeal cancer tissue and non-cancerous laryngeal mucosa homogenates were probed with the RL2 anti-*O*-GlcNAc antibody where multiple bands were detected and used to quantify protein *O*-GlcNAcylation. The average values of *O*-GlcNAc levels demonstrated significant increases of ~60 % in total protein *O*-GlcNAcylation levels in SCLC compared with NCLM (*p* < 0.01). Moreover, hyper-*O*-GlcNAc levels for proteins of approximately 97 kDa molecular mass in SCLC were observed, as well as any differences between SCLC and NCLM with regard to *O*-GlcNAcylation profile for proteins with molecular mass beneath 40 kDa. A representative image obtained from NCLM and SCLC sample homogenates probed with the RL2 antibody and plots with the average values of total protein *O*-GlcNAcylation levels are shown in Fig. 3.

### Association between *OGT* and *MGEA5* transcripts and clinicopathological characteristics in laryngeal cancer

**Fig. 4:**
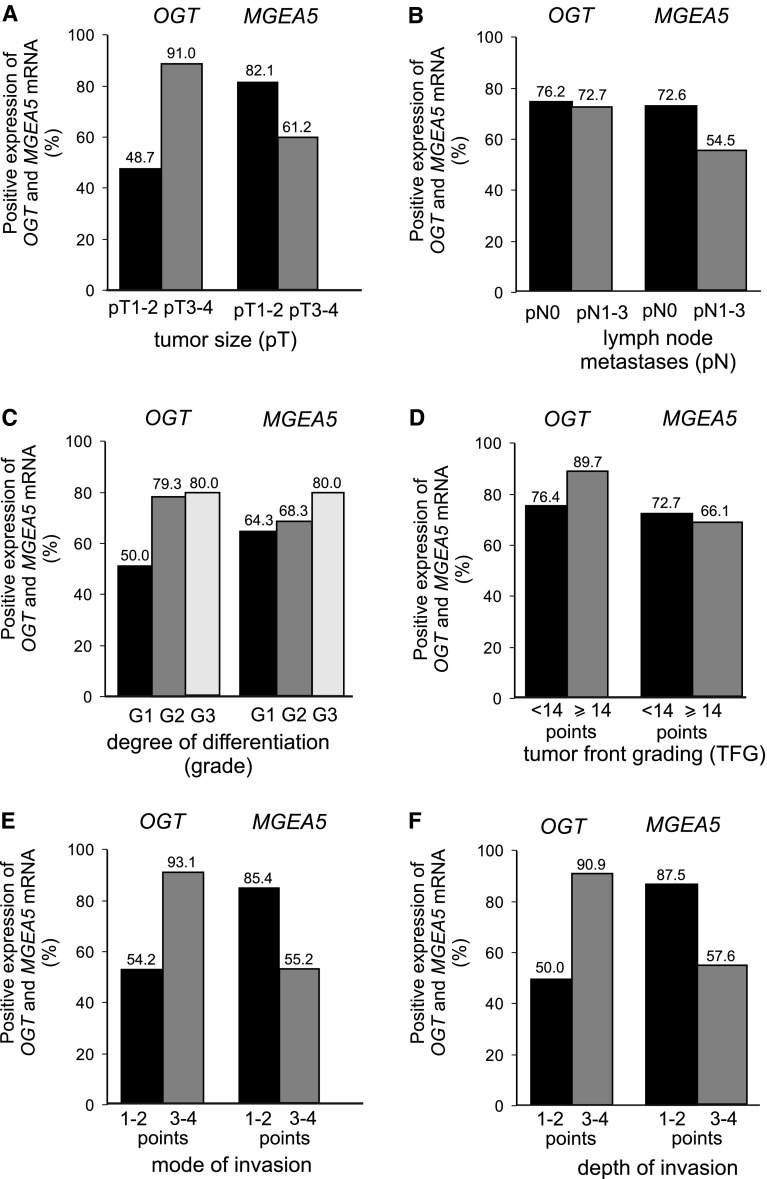
Positive expressions of *OGT* and *MGEA5* transcripts (%) in laryngeal cancers; a relation to the relevant clinicopathological characteristics: Tumor extension (pT) (**a**), cervical lymph node metastases (pN) (**b**), degree of differentiation (grade) (**c**), total score of tumor front grading (**d**), mode (**e**) and depth of invasion (**f**)

This study also investigates whether *OGT* and *MGEA5* transcripts may affect tumor behavior. The relationships between *O*-GlcNAc transferase and *O*-GlcNAcase gene expression and the relevant clinicopathological characteristics in laryngeal cancers, are shown in Fig. 4. The mRNA *OGT* level in laryngeal cancer tissue was significantly related to local tumor extension (pT) and nodal metastases (pN) (*p* < 0.0001 and *p* < 0.0001, respectively) as well as degree of histological differentiation (grade) (*p* = 0.003). The presence of higher levels of *OGT* transcripts in SCLC was associated with a larger tumor size, more frequent incidence of cervical lymph node metastases and higher histological grade. Significant differences were also noted in the expression of the *O*-GlcNAc transferase gene between grade 3 and grade 1 tumors (*p* < 0.001) as well as between grade 3 and grade 2 cancers (*p* < 0.001). A similar significant positive association was determined concerning total TFG score (*p* < 0.0001) as well as mode and depth of invasion (*p* < 0.0001 and *p* < 0.001, respectively). The presence of increased levels of *OGT* transcript in tumor tissue was more frequently associated with greater total TFG score (≥14 points) as well as deeper and more aggressive tumor growth with submucosa or cartilage infiltration and disseminated type of invasion.

**Fig. 5:**
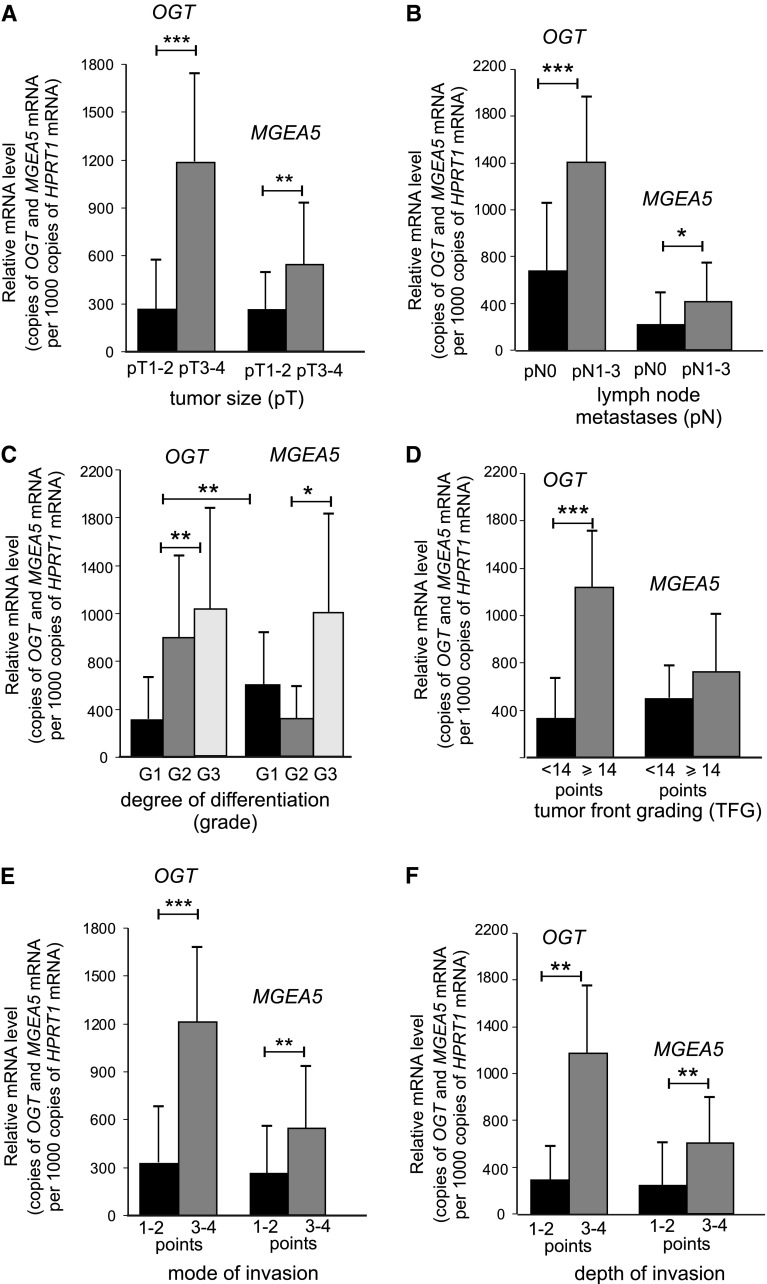
Expression of mean *OGT* and *MGEA5* mRNA measured by real-time PCR in laryngeal cancers; a comparison between subgroups with local tumor extension (pT) (**a**), cervical lymph node metastases (pN) (**b**), degree of differentiation (grade) (**c**), total score of tumor front grading (**d**), mode (**e**) and depth of invasion (**f**). *Graphs* represent mean ± standard deviation; *p < 0.005, **p < 0.001, ***p < 0.0001

Similarly, an increased *MGEA5* mRNA level in SCLC was related to higher pT and pN status of laryngeal cancer cases (*p* < 0.0001 and *p* = 0.013, respectively). Moreover, *MGEA5* mRNA expression was higher in grade 3 than grade 1 or 2 tumors. A significant difference in expression of *O*-GlcNAcase gene was observed between grade 3 and grade 2 cancers (*p* < 0.005). In addition, relationships were noted between *MGEA5* gene level and both type and depth of invasion (*p* = 0.001 and *p* = 0.001, respectively). A higher expression of *MGEA5* transcripts was observed for SCLC characterized by submucosa or cartilage infiltration and diffuse mode of tumor growth. The association between clinicopathological parameters and both *OGT* and *MGEA5* transcript expressions and the results of the statistical analysis are shown in Fig. 5. as well as the results of the statistical analysis.

**Fig. 6:**
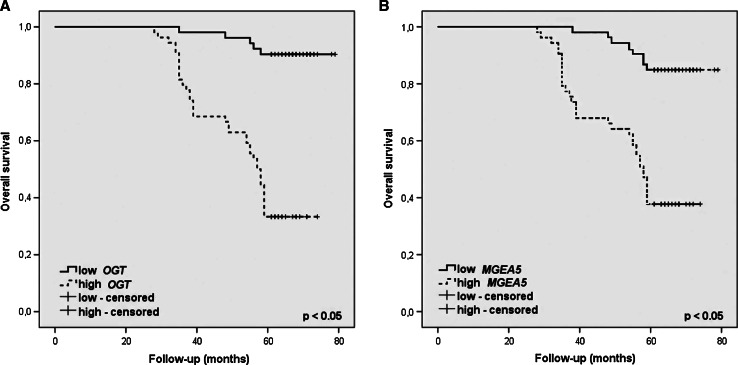
Kaplan–Meier plots of overall survival for categorized by *OGT* (**a**) and *MGEA5* (**b**) mRNA expression in homogenate samples of laryngeal cancer tissues; the cutoff value was established to be the median of *OGT* and *MGEA5* mRNA levels. The survival curves were compared between two groups: High (≥median value) and low (<median value) expression

The present study also investigated whether *OGT* and *MGEA5* transcripts level can potentially determine a prognosis for the patient. Interestingly, the expression of both *O*-GlcNAc-cycling enzyme genes was found to be significantly related to incidences of recurrences and 5-year overall survival. An increased level of mRNA *OGT* was shown to promote the presence of both local and nodal recurrences (*p* < 0.001 and *p* = 0.0008) as well as the survival time of <5 years (*p* = 0.01). A similar case was observed regarding the relationship between *MGEA5* mRNA level and incidence of nodal recurrences (*p* = 0.03) and 5-year survival (*p* = 0.0001). Moreover, overall survival was analyzed through the Kaplan–Meier plots as shown in Fig. 6. In survival analysis of *OGT* gene level, there were 52 (49.1 %) cases of low and 54 (50.9 %) cases of high *OGT* mRNA expression. Mean overall survival time was 76.3 and 55.4 months in low and high *OGT* expression groups, respectively. High expression of *OGT* gene level showed worse overall survival rate than those of low expression one (*p* < 0.0001). In survival analysis of *MGEA5* transcripts level, there were 50 % of patients in both groups of low and high *MGEA5* mRNA expression. Mean overall survival time was 74.9 months in low *MGEA5* mRNA expression and 56.1 months in high *MGEA5* expression. High level of *MGEA5* transcripts showed poorer overall survival rate than low expression group with significant difference (*p* < 0.0001).

### Association between OGT and OGA proteins and clinicopathological characteristics in laryngeal cancer

The next stage also investigated whether OGT and OGA proteins can potentially determine the pTNM classification features of laryngeal cancer, since the tumor stage is currently the only accepted prognostic marker. As a result, a positive expression of OGT protein was noticed in half the samples (5/10) of poorly differentiated laryngeal cancer (grade 3), 58.3 % (7/12) of moderately differentiated tumors (grade 2) and 20 % (2/10) of well-differentiated tumors (grade 1). The mean expression of *O*-GlcNAc transferase in neoplastic tissue was higher for tumors with a higher degree of histological differentiation (*p* = 0.027). Similarly, the expression of OGA protein was noticed in 80 % (8/10) of poorly differentiated laryngeal cancers (grade 3), 58.3 % (7/12) of moderately differentiated tumors (grade 2) and 40 % (4/10) of well-differentiated (grade 1) laryngeal cancers. The presence of a higher level of *O*-GlcNAcase in tumor samples was also associated with a higher tumor histological differentiation (*p* = 0.047). Statistically significant differences in OGT protein expression were observed between grade 3 and grade 1 as well as between grade 3 and grade 2 tumors (*p* = 0.021 and *p* = 0.038, respectively) as well as in OGA protein level between grade 3 and grade 1, as well as grade 2 and grade 1 cancers (*p* = 0.016 and *p* = 0.036, respectively). The results concerning OGT and OGA expressions in homogenate samples of laryngeal cancer tissues in relation to tumor grade are shown in Fig. 1. Of 32 laryngeal cancer samples, OGT protein-positive expression was noticed in 47.1 % (8/17) and 40 % (6/15) tumors classified according to pTNM as pT3–pT4 and pT1–pT2, respectively. Of the laryngeal cancer samples, 5 of the 22 pN0 tumors (22.7 %) and 9 of the 10 pN1–3 tumors (90 %) demonstrated positive expression of OGT. Subsequently, 53.3 % (8/15) pT1–pT2 tumors and 64.7 % (11/17) pT3–pT4 cancers were found to be positive for OGA. Nine of the 22 pN0 laryngeal cancer samples (40.9 %) and all 10 (100 %) of the pN1–3 samples demonstrated positive expression of OGA. Unfortunately, no significant correlation was found between either OGT or OGA expression and the relevant features (*p* > 0.05). However, it should be emphasized that more advanced tumors (pT3–pT4, N1–3) were more commonly found to be positive in a quantitative densitometric analysis, and to have higher mean OGT and OGA protein levels, than less advanced laryngeal cancers.

## Discussion

*O*-linked β-*N*-acetylglucosamine is a regulatory posttranslational modification (*O*-GlcNAcylation) of intracellular cytoplasmic, nuclear and mitochondrial proteins essential for fundamental cellular processes such as transcription, translation, signal transduction, nuclear transport, protein stability and protein–protein interaction [1–4]. Aberrant protein *O*-GlcNAcylation may contribute to pathological mechanisms of various disorders, as well as to the development, malignant behavior and progression of many cancers [1, 5–9]. In the last half decade, a growing body of evidence indicates that this PTM plays a crucial role in carcinogenesis and regulates tumor metabolic reprogramming by modifying key transcription factors, metabolic enzymes and oncogenic signaling pathways. However, the direct functional connections and an understanding of the specific mechanisms through which *O*-GlcNAc influences cancer cell dynamics and pathology have remained elusive [5–9].

The aim of the present study was to analyze the gene and protein expression of *O*-GlcNAc transferase and *O*-GlcNAcase as well as total protein *O*-GlcNAcylation levels in cancer tissue compared with matched adjacent tissue, and to determine whether OGT and OGA can promote the invasion of squamous cell laryngeal cancer and affect patient prognosis. This is the first study to identify *O*-GlcNAc total levels in laryngeal cancer tissue and normal laryngeal mucosa, as well as an association between *O*-GlcNAc-cycling enzymes and the dynamics of tumor growth and aggressiveness in this type of cancer. Most importantly, the analyzed material constitutes a large, homogeneous group of head and neck cancers sharing the same origin. Our research is valuable and outstanding in this regard, considering the difficulties in obtaining the fresh human biopsy tissues such as cancerous and non-cancerous normal laryngeal mucosa.

In the present study, higher levels of both OGT and OGA proteins were noted in neoplastic tissue than in the adjacent normal laryngeal mucosa. Moreover, the evaluation of *O*-GlcNAc levels demonstrated significant increases of ~60 % in total protein *O*-GlcNAcylation levels in cancer tissue compared with matched adjacent tissue.

Unfortunately, a literature review revealed no publication describing the expression of *O*-GlcNAc-cycling enzymes and total *O*-GlcNAc levels in malignant head and neck tumors. However, our findings resemble the findings in other cancers, particularly with regard to the OGT enzyme, regardless of tumor type [11, 14, 20–28]. For instance, Phueaouan et al. [23] demonstrated increased *O*-GlcNAcylation in primary colorectal cancer tissues associated with overexpression of OGT. A similar result was reported by Itkonen et al. [11] for prostate cancer in which upregulation at the protein level of *O*-GlcNAc transferase was established. Similarly, Champattanachai et al. [31] identified a positive association between some tumor-associated *O*-GlcNAc-modified proteins with elevated expression of OGT in primary breast cancer. However, the literature survey more often shows divergent results concerning *O*-GlcNAcase in cancers. For example, Krześlak et al. [27], in a study regarding the expression of genes encoding *O*-GlcNAc-cycling enzymes in endometrial carcinoma, noted that both *OGT* and *MGEA5* mRNA expressions were found to be significantly higher in tumor tissue, as is found in the present study. By contrast, Ma et al. [14] observed that hyper-*O*-GlcNAcylation in pancreatic ductal adenocarcinoma was associated with an increased level of OGT, but a reduction in OGA activity. Similarly, Phoomak et al. [32] reported that OGT and OGA levels in bile duct carcinoma tissues and the expression of *O*-GlcNAc-modified proteins, correspond to the upregulation of *O*-GlcNAc transferase and downregulation of *O*-GlcNAcase. Enhanced *O*-GlcNAcylation and low OGA protein expression were also determined by Zhu et al. [20] in hepatocellular carcinoma.

It should be emphasized that in our study, hyper-*O*-GlcNAcylation levels in laryngeal cancer tissue compared with normal mucosa was associated with an increased level of both OGT and OGA proteins. The presence of *O*-GlcNAc overexpression in spite of an increased level of antagonistic *O*-GlcNAc-cycling enzymes requires further explanation. It is important to understand that *O*-GlcNAc-cycling enzyme activity cannot be balanced, and the final and total protein *O*-GlcNAcylation levels are not based on a simple mathematical subtraction of enzyme expression. While the domain structures of OGT and OGA, as well as those in complexes with substrates, are widely discussed in the literature, the regulation of their substrate specificity, the catalytic mechanisms determining the extent to which substrates are modified by *O*-GlcNAc-cycling enzymes, as well as the role of their modulators, the formation of OGT/OGA complexes and molecular function of *O*-GlcNAc process still remain to be discovered.

Hence, hyper-*O*-GlcNAcylation, which has been confirmed by other researchers in various neoplastic diseases, may be due to several reasons, independent of the observed expression of *O*-GlcNAc-cycling enzymes [11, 14, 20–28]. One possible reason is that OGT and OGA act on hundreds of different protein substrates to regulate *O*-linked β-d-*N*-acetylglucosamine levels at different sites, which implies that the inducible cycling of this modification is not parallel and simultaneously managed by these two enzymes [33]. Most importantly, the expressions of OGT and OGA are not equivalent to their activity. The regulation of *O*-GlcNAcase activity has been proposed to appear via posttranslational modification, various regulatory protein–protein interactions modulating substrate specificity (which may not enhance, but impair catalysis by OGA), and the occurrence of different structural variants of OGA, which also vary in terms of their activity on both proteins and synthetic substrates [33, 34]. Moreover, OGA isoforms have other cellular factors and different protein partners required for their full activity [33, 35]. It is not without significance that formation of an OGT/OGA complex that controls OGA isoform activity may affect different target proteins. Accordingly, the ratio of OGT to OGA can influence the extent of *O*-GlcNAcylation levels in study tissues [36]. Also, *O*-GlcNAc transferase function could be regulated by posttranslational modification and *O*-GlcNAc modification. It is well known that *O*-GlcNAc levels respond to various stimuli, including cellular stress and cell-cycle progression, which thus regulate and influence the final modification of *O*-GlcNAc, regardless of the OGA activity [37]. Also, research on human biopsy material and analysis of human OGA (hOGA) expression remains as a valuable complement to the results obtained in other research on bacterial homologues of hOGA (the differences between cellular and in vitro system).

It should be also added that the antibodies used by researchers to identify *O*-GlcNAc are various in terms of specificity and profile, and thus may not detect all modified proteins, leading to divergent results with regard to total *O*-GlcNAcylation levels in various types of cancers [33]. Moreover, the marked increased *O*-GlcNAc levels in laryngeal cancer homogenates for proteins of 97 kDa molecular mass were not observed in matched adjacent tissue, as well as the differences in protein *O*-GlcNAcylation profile for proteins with molecular mass below 40 kDa in tumor samples compared with normal mucosa, should be taken into consideration in the interpretation of hyper-*O*-GlcNAcylation levels. This specificity changes in protein *O*-GlcNAcylation levels in this type of head and neck neoplasm may also affect the overall protein *O*-GlcNAc content.

Despite the divergent findings regarding levels of OGT and OGA enzymes in cancers, the results of research have confirmed the carcinogenic potential of *O*-GlcNAc-cycling enzymes in tumor development and progression [11, 14, 20–28]. Most studies report increased protein *O*-GlcNAcylation and the presence of changes in *OGT* and/or *OGA* mRNA and protein expression in prostate, colorectal, pancreatic, breast, ovary, urinary bladder, bile duct, endometrial, lung or liver cancers, where a positive association was disclosed between *O*-GlcNAc-cycling enzyme expression and a number of clinicopathological variables including higher tumor grade, stage, higher differentiation, enhanced tumor cell proliferation and invasion as well as poor prognosis [11, 14, 20–28]. However, other studies note that *O*-GlcNAc-cycling enzyme expression was either inversely correlated or unrelated to tumor progression [17, 27, 28].

In our study, both *OGT* and *MGEA5* transcript levels were significantly related to cancer invasiveness. As a result, increased levels of *OGT* and *MGEA5* mRNA were found to be related to larger tumor size, nodal metastases, higher grade and tumor behavior according to the TFG scale, as well as incidence of disease recurrences. An inverse association between *OGT* and *MGEA5* transcripts was determined with regard to prognosis. In addition, the highest OGT and OGA protein levels were observed in poorly differentiated tumors. No correlations with other parameters were noted, but the results showed a trend of more advanced tumors to be more frequently OGT and OGA positive.

Unfortunately, no data concerning the relationship of OGT and/or OGA (*MGEA5*) with the presence of an invasive tumor phenotype in malignant head and neck tumors could be found in the literature. It should be stressed that in all studies concerning *O*-GlcNAc-cycling enzymes, their expression in cancer progression was only examined with respect to such clinical parameters as primary tumor size, nodal status, stage, histological grade of malignancy and prognosis. In the present study, the clinicopathological evaluation considers the morphological tumor front grading (TFG), which includes a precise evaluation of such tumor-related features and stroma-related characteristics of the peripheral edge of tumor infiltration as mode and depth of invasion. Regardless of these factors, the resulting data indicate that aberrant protein *O*-GlcNAcylation may have an effect on tumor aggressiveness in laryngeal cancer and these observations are consistent with the results regarding other cancers. For instance, Kamigaito et al. [21] reported that overexpression of *O*-GlcNAc in neoplastic tissue exerts tumor cell activity and that it may be significant in the determination of aggressive behavior of prostate cancer. The authors reveal a positive association between the cellular distribution of *O*-GlcNAc and clinicomorphological features such as enhanced tumor cell proliferation, grade of invasion and poor prognosis, and concluded that increased *O*-GlcNAc tissue level may be implicated in the development and progression of prostate cancer. Similar results for OGT immunohistochemical overexpression relating to such clinical parameters as larger primary tumor size, positive nodal status, higher grade of malignancy according to Gleason scale and increased metastases incidence were also reported in another study of prostate cancer. Itkonen et al. [11] noted an increase in *O*-linked β-*N*-acetylglucosamine transferase expression in cancer cells, and suggested that OGT can affect c-Myc stability, and thus may be related to cell-cycle progression and DNA. The authors observed that RNA interference-mediated silencing or pharmacologic inhibition of *OGT* correlated with prostate cancer cell growth. In another study, Jin et al. [38] also reported an elevated *O*-GlcNAcylation level in an ovarian cancer model and its association with tumor cell migration. The authors suggested that increased *O*-GlcNAc modification by *OGT* silencing or *OGA* inhibition in tumor cells may be associated with decreased E-cadherin expression and may also be linked with higher incidence of metastases in the study model. Also, Yehezkel et al. [25] revealed that *O*-GlcNAcylation level in colorectal cancer clones corresponds to phenotypic alterations promoting epithelial metastatic progression and tumor growth. Różański et al. [24] noted that the positive expression of *MGEA5* mRNA was found in the urine of both healthy persons and bladder cancer patients, but *OGT* mRNA was not found in healthy individuals. The authors observed that poorly differentiated cancers demonstrated higher *O*-linked β-*N*-acetylglucosamine transferase expression and lower *MGEA5* levels were present in patient urine. Moreover, a significant difference in *OGT* mRNA expression was observed between early and invasive bladder cancers. A similar result was reported by Krześlak et al. [27] for endometrial cancer in which the *OGT* and *OGA* mRNA expressions were significantly increased in tumors of higher histological grade and deeper invasion of myometrium. However, both enzyme mRNA levels were unrelated to clinical disease stage. Lynch et al. [19] also confirmed a crucial role of *O*-linked β-*N*-acetylglucosamine transferase in invasion, metastasis and angiogenesis in prostate cancer cells lines, insofar that regulation of malignant properties were found to be connected with higher OGT protein and *O*-GlcNAcylation levels. Moreover, the authors noted that the reduction in OGT expression in tumor cells was associated with decreased expression of MMP-2, MMP-9 and VEGF and blocked bone metastasis. Similar results were reported by Zhu et al. [20], who suggested that *O*-GlcNAcase level may be an independent prognostic factor for predicting tumor recurrence in hepatocellular carcinoma, and that *O*-GlcNAcylation plays an important role in migration, invasion of HCC cells through regulating E-cadherin and MMP expression. Overexpressions of *O*-GlcNAc-modified proteins and OGT were also observed by Phoomak et al. [32] in cholangiocarcinoma. In this case, elevated immunohistochemical expression of *O*-GlcNAcylation was found to be significantly higher in tumor tissues and was associated with a poor patient outcome.

It should be emphasized that there are several constraints in interpreting results concerning the OGT and/or OGA activity in tumorigenesis and cancer progression. While *O*-GlcNAc-cycling enzymes are presented in most of the publications as useful potential biomarkers for tumor behavior in various cancers, discrepancies exist due to differences in their biology caused by variation of tumor types, histological differentiation status and proliferative index. Also, the choice to base the research on materials from cell culture or laboratory animals, due to the difficulties in obtaining human autopsy materials and the diversity of tissues used (fresh tumor samples, paraffin-embed samples, isolated leukocytes or blood), may have an impact on the results. Moreover, the relatively small size of the study group can influence the resulting data. Despite these limitations, the findings nevertheless illustrate the importance of *O*-linked β-*N*-acetylglucosamine posttranslational modification in inducing a malignant phenotype of cancer cells of various origins and provide possible targets for diagnosis and a forthcoming new perspective on cancer treatment. A future challenge will be to use the knowledge of the mechanisms of *O*-linked β-*N*-acetylglucosamine posttranslational modification of oncogenic-related proteins and crucial signaling pathways to propose novel strategies for inhibition of carcinogenesis, tumor growth and cancer progression.

## Conclusions

In conclusion, our results suggest that the abnormalities in *O*-GlcNAc-cycling enzymes leading to the aberrant protein *O*-GlcNAcylation could be considered as new biomarkers of the invasive phenotype of tumor cells and prognosis and perhaps a potential new approach for laryngeal cancer treatment. In this context, further studies of the precise roles of OGT and OGA and understanding the regulatory mechanisms occurring in laryngeal cancer will be needed.
